# The effects of Medicaid expansion on the racial/ethnic composition within nursing home residents

**DOI:** 10.1186/s13561-024-00517-3

**Published:** 2024-06-20

**Authors:** Fernando Loaiza

**Affiliations:** Max Planck Institute for Social Law and Social Policy, Amalienstraße 33, 80799 Munich, Germany

**Keywords:** Medicaid expansion, Affordable Care Act, Race, Nursing home, H51, I11, I13, I14, I18, I38

## Abstract

**Background:**

The Affordable Care Act (ACA), enacted in 2010, aimed to improve healthcare coverage for American citizens. This study investigates the impact of Medicaid expansion (ME) under the ACA on the racial and ethnic composition of nursing home admissions in the U.S., focusing on whether ME has led to increased representation of racial/ethnic minorities in nursing homes.

**Methods:**

A difference-in-differences estimation methodology was employed, using U.S. county-level aggregate data from 2000 to 2019. This approach accounted for multiple time periods and variations in treatment timing to analyze changes in the racial and ethnic composition of nursing home admissions post-ME. Additionally, two-way fixed effects (TWFE) regression was utilized to enhance robustness and validate the findings.

**Results:**

The analysis revealed that the racial and ethnic composition of nursing home admissions has become more homogeneous following Medicaid expansion. Specifically, there was a decline in Black residents and an increase in White residents in nursing homes. Additionally, significant differences were found when categorizing states by income inequality, and poverty rate levels. These findings remain statistically significant even after controlling for additional variables, indicating that ME influences the racial makeup of nursing home admissions.

**Conclusions:**

Medicaid expansion has not diversified nursing home demographics as hypothesized; instead, it has led to a more uniform racial composition, favoring White residents. This trend may be driven by nursing home preferences and financial incentives, which could favor residents with private insurance or higher personal funds. Mechanisms such as payment preferences and local cost variations likely contribute to these shifts, potentially disadvantaging Medicaid-reliant minority residents. These findings highlight the complex interplay between healthcare policy implementation and racial disparities in access to long-term care, suggesting a need for further research on the underlying mechanisms and implications for policy refinement.

**Supplementary Information:**

The online version contains supplementary material available at 10.1186/s13561-024-00517-3.

## Introduction

Long-term care is becoming an essential challenge to governments of developed countries as the population share of individuals aged 85 and older in the EU27 is expected to double over the next 30 years. The baby boomers are approaching retirement and there is the possibility that their long-term care needs will not be fully covered. A central issue regarding care needs for the elderly is that they are expensive, and most of the population does not have the means to afford them. In the U.S., healthcare coverage is publicly and privately provided. However, this coverage is not universal, and it is even more restricted when related to long-term care services. If indeed the number of people requiring such services increases due to the big population share that involves baby boomers, unmet long-term care needs will likely increase too. This raises concerns about an exacerbation of current health and economic problems among the poor in the future. Previously, to address this issue of affordability and coverage for health care, programs such as Medicaid were implemented. Even though it provides support with healthcare-related expenses for people with limited resources, it still has several flaws. As we consider the implications of an aging population and the expanding need for care, additional challenges emerge. First is the existing healthcare system’s capacity to accommodate the increase in demand from beneficiaries where the supply of healthcare professionals may struggle to keep pace [1]. Second, is the enrollment process itself, which, due to its complexity, could deter the very individuals Medicaid aims to assist [2]. These barriers, while not the primary focus of Medicaid’s implementation, underscore the multifaceted nature of healthcare access.

The ACA, signed into law in 2010 and majorly implemented in 2014, represents a significant policy aimed at addressing these challenges. As of now, thirty-nine states and the District of Columbia have adopted the ACA, which includes a provision for Medicaid expansion (ME). This expansion aims to extend coverage to individuals earning less than 138% of the federal poverty line, with the federal government bearing most costs and states contributing incrementally over time. However, the high costs of nursing home care-a service not typically covered under essential health benefits-exacerbate long-term care challenges. Medicaid, though a primary contributor to nursing home expenses, is means-tested and has limitations, such as the small personal needs allowance for beneficiaries. Despite Medicaid’s 100% coverage for eligible individuals in approved facilities, coverage issues persist due to a preference for privately paying residents in nursing homes.

In this study, I delve into the impact of ME on nursing home admissions across various racial and ethnic groups. The motivation for this exploration is twofold. Firstly, there’s an escalating concern about the economic racial differences in the U.S., particularly as Black and Hispanic communities represent a disproportionate share of the impoverished population as shown in Figure 1 in the Appendix. This overrepresentation of these communities in lower economic strata suggests that ME could significantly alter the racial and ethnic composition of nursing home populations. By expanding healthcare coverage to include a broader segment of the low-income population, ME has the inherent potential to directly impact those most affected by health disparities, thereby influencing the demographic composition of nursing homes. Secondly, there’s a looming challenge regarding the affordability and accessibility of long-term care services, both presently and in the foreseeable future. Given the historical barriers faced by racial and ethnic minorities in accessing healthcare services, including long-term care, ME’s role in potentially mitigating these barriers is of great importance. The ME aims to bridge the gap in healthcare access, making long-term care services more attainable for economically disadvantaged minorities.

This study, therefore, seeks to understand how ME, as a policy intervention, impacts nursing home admissions among racial and ethnic groups, against the backdrop of these issues. By examining the changes in the racial and ethnic composition of nursing home populations post-ME, this research aims to shed light on the policy’s effectiveness in addressing long-standing disparities and the extent to which it contributes to more inclusive healthcare access. The primary hypothesis of this study is that ME is designed to enhance healthcare coverage for the impoverished, and thus, we should expect an increase in nursing home admissions from these communities, including racial and ethnic minorities who are disproportionately represented among the economically disadvantaged. Conversely, an equally plausible alternative hypothesis posits that the persistent preference for privately paying residents within nursing homes, coupled with the scarcity of available space, may lead to a competitive disadvantage for Medicaid-insured residents, potentially resulting in a decrease in admissions for minority residents who are more likely to be covered by Medicaid after ME. In considering both hypotheses, the study seeks to provide a comprehensive analysis of ME’s outcomes. By examining both the surge in admissions due to expanded coverage and the potential decrease due to systemic preferences within nursing homes, the research can offer a nuanced understanding of ME’s impact on the racial and ethnic composition of nursing home populations.

Employing Andersen’s Behavioral Model of Health Services Use as a theoretical backdrop [3, 4], the expansion of Medicaid, by altering individual characteristics (increased eligibility), external conditions (service availability), and perceptions of care as a viable option, may influence the racial and ethnic composition of nursing home admissions. Under Andersen’s Behavioral Model, it would seem likely that the ME would increase the proportion of individuals from under-served groups admitted to nursing homes.

There might be several pathways through which ME is anticipated to affect the racial/ethnic composition of nursing home admissions. Besides the direct path that is increasing access for previously under-insured impoverished groups, there are two alternatives discussed here. ME may result in shifts in the geographic distribution of nursing home admissions. Areas with higher concentrations of racial and ethnic minorities, which may also correspond with regions of higher poverty levels, could experience more substantial increases in admissions post-expansion. Another possibility is that changes in healthcare providers might alter their admission practices in response to ME, potentially affecting the racial/ethnic composition of their nursing home resident populations.

Projections from the Genworth Cost of Care Survey indicate that the average annual cost for a semi-private nursing home room in the U.S. was $94,900 in 2020. Meanwhile, the U.S. Census Bureau reported a median household income of $67,521 in 2020, highlighting the affordability gap in nursing home care, particularly for over half of the population, and even more for Black and Hispanic communities with typically lower incomes. This economic stratification by race/ethnicity, with White individuals generally having higher incomes [5], underscores the paper’s research question: based on the high costs of nursing home services and the different poverty rates by race/ethnicity in the U.S., how has ME influenced the racial/ethnic composition of nursing home admissions? To address this, I implement a new difference-in-differences (DiD) estimation methodology introduced by [6] to obtain accurate causal effects arising from the policy enactment.

A growing literature examines the general effects of the ME and found positive effects on insurance coverage, health outcomes, and access and use of care services [7–12], admission to mental health treatment [13], reduction on mortality rates [14], foster care admissions [15], unpaid bills, and the amount of debt sent to third-party collection agencies [16]. Negative effects are also reported, for example, longer waiting times for appointments [17], cost-related barriers for senior citizens, delaying care, paying drug prescriptions, less access to specialist doctors, or lack of continuity of care for cost reasons [18]. Moreover, [19] highlights persistent disparities in specific healthcare areas, such as oral health services for adults with disabilities in rural areas. Despite slight overall improvements after the policy implementation, significant gaps in preventive oral care access remain, underlining the necessity of targeted policies to address health disparities and improve equitable healthcare access.

A smaller amount of research has been developed to understand the impact of Medicaid expansion by race. Reduction in uninsured rates were found by [18, 20–22]. However, [12] found no significant evidence of a reduction of racial differences in insurance coverage for adults below the poverty line and adults without children. Additionally, different studies found race-related effects regarding the quality of care [23] and consistent source of care, unmet needs of care due to cost, or mental health [24]. In general, several authors indicate a reduction of racial differences in health insurance coverage; however many other areas still show high levels of disparity and a differential impact on coverage and services for different races or ethnicities.

The literature, regardless of racial differences, on long-term care services and ME, is even smaller. The first evidence according to [25] is that for newly eligible individuals there is an increase in any long-term care use suggesting that before the expansion, there were a high amount of long-term care unmet needs. In general, the literature suggests that historically racial segregation in health care services remains high [26, 27] including nursing homes [28]. The findings of [28], obtained by using the Dissimilarity Index between Black and White residents, not only suggest that racial segregation remains high in nursing homes within metropolitan areas but more importantly argue that the quality of care that racial minorities receive is lower. Even though their research is not about the ME effects on nursing home composition, it still shows the historical segregation in nursing homes in the year 2000. According to [29], distance and especially race-based preferences contribute to an unequal racial composition in nursing homes. This means that Black individuals who live in predominantly White areas travel farther to go to nursing homes with a bigger share of Black residents regardless of the lower quality of care. In a detailed review of the vast literature on racial segregation in nursing homes, [30] concludes this remains an extensive problem for all the different measures of segregation.

The results of this research contribute to the literature on the benefits of ME but more specifically is a new contribution to the smaller area of racial or ethnic composition of nursing home residents. This research complements [28] findings, although with a different approach, by including the evolution of nursing home admissions by race and ethnicity and the role of the ME. The results, obtained by using aggregate data at the U.S. County level from 2000 to 2019, indicate that the racial and ethnic composition of nursing home residents is not showing improved representation for minorities; instead, it is becoming more homogeneous. This is due to a reduction in the aggregate number of Black residents and the increase of White residents in nursing homes after the expansion of Medicaid. This exacerbates the unevenness of the racial/ethnic composition of the residents of nursing homes. To further understand these results, the effects of the ME are also analyzed by classifying states by poverty rate, and income inequality. It is argued that a potential mechanism for explaining these results is the combination of a reduction in the total number of beds available for Medicaid patients and the increase of private ones in nursing home facilities. The remainder of the paper is organized as follows. Methods section details of the empirical strategy and data. Results section shows the results of the analysis and the potential mechanisms driving these results. Discussion section discusses the findings and Conclusions section concludes.

## Methods

In response to the varied timing of Medicaid Expansion across states, this paper uses a Difference-in-Differences (DiD) approach as outlined by [6] (CS), combined with two-way fixed effect (TWFE) regression, to analyze the impact of Medicaid Expansion on the racial and ethnic composition of nursing home admissions. I include counties that belong to states that are classified as never-treated (states that did not expand Medicaid or expand after 2019), not-yet-treated (states that passed the law after 2014), or treated (states that passed the law in 2014). This means that different groups of counties are exposed to the policy at different times. The Callaway and Sant’Anna method employs various aggregation schemes to explicitly accommodate the staggered nature of policy implementation, overcoming limitations identified in other methodologies when dealing with heterogeneous treatment effects and varying treatment timing [31–35]. These schemes are designed to take into account the variations in both the timing of policy adoption and the duration of treatment, thus improving the analysis of dynamic treatment effects and diminishing estimation uncertainty. For brevity, while multiple aggregation schemes will initially be presented to summarize the main results, the focus will mostly be on one scheme if the results align. To refine the analysis, this study employs doubly robust (DR) estimators for more accurate average treatment effects on the treated (ATT). The CS approach, alongside the TWFE regression, aims to provide reliable insights into how different years of ME implementation in various states affect nursing home admissions by race/ethnicity. The model specification of the TWFE follows:

1 $$\begin{aligned} Y_{ct} = \alpha _{z} + \gamma _{t} + \beta ME_{ct} + X_{ct} + \epsilon _{ct} , \end{aligned}$$

where $Y_{ct}$ is the admissions to nursing homes by race/ethnicity in a year *t*. $ME_{ct}$ is an indicator variable that is equal to 1 if the Medicaid expansion is implemented in a county *c* (belonging to the state that expanded Medicaid.), $X_{ct}$ is a matrix of control variables. We include county fixed effects $\alpha _{z}$ and $\gamma _{t}$, to capture time-invariant geographical unobservables. Additionally, $\epsilon _{ct}$, to account for unobserved confounders.

This study analyzes county-level panel data from 2000 to 2019, focusing on the racial and ethnic composition of nursing home admissions in the U.S., using data from LTCFocus at Brown University and the National Institute on Aging. The dependent variables will characterize the racial/ethnic composition of residents admitted to nursing homes during a calendar year in a specific county. This composition provides the share of individuals who are ‘Black, not of Hispanic origin’, ‘Hispanic’, and ‘White, not of Hispanic origin’ at the county level. Additional control variables are also introduced. The occupancy rate shows the number of occupied beds divided by the total number of beds. Nursing home (N.H.) concentration measures the competition in a county ranging from 0 to 1. A county with a concentration level close to 1 has a monopoly on nursing home beds. Lastly, the “For-Profit” variable provides information about the type of facility including whether it is for-profit or not. This variable shows the percentage of facilities in a particular county that are for-profit. Income per capita was included in the analysis and was obtained from the U.S. Department of Commerce and the total population and population by race was obtained from the United States Census Bureau. Political Preference is a variable categorizing counties, based on the winning party in the most recent elections in the respective state [36]. It assigns a value of 1 for states won by the Democratic Party and 2 for those won by the Republican Party. This variable is updated to reflect the outcomes of each state’s elections, ensuring its relevance and accuracy over time. Lastly, variables controlling for the White and Black male population are included.

The year of the ME by state is provided in Table A1. As the panel data used in this analysis is from the year 2000 to 2019, states that expanded Medicaid after 2019 appear as non-expansion states. Figure 2 shows the number of states and counties by the year that they expanded Medicaid. In this figure, the first bar “0” shows the number of states considered as “not-treated” i.e., states that never expanded Medicaid or states with the implementation after 2019. Interestingly, most expansion states implemented the provisions in 2014. A correlation matrix and summary statistics are presented in Tables A2 and A3 respectively in the Appendix. Among the most important things to mention is that the composition of the nursing home residents by race/ethnicity is very unequal with 87% of them being White. This unevenness among nursing home residents can be observed also in Figure A3 which shows the composition of nursing home facilities for expansion and non-expansion states before and after the expansion of Medicaid in 2014. Interestingly, the increase (decrease) of admissions of Black (White) residents before and after the implementation of the policy, can be observed in both the expansion and non-expansion states. Another interesting insight obtained from this descriptive analysis is that the primary support for the biggest share of residents in nursing home facilities is Medicaid, accounting for around 65% of the total. Conversely and even lower than private residents, Medicare accounts only for around 10% of the support.

Table A4 presents a detailed summary of our analysis, comparing key variables between groups subjected to treatment and those that were not. Interestingly, the statistics reveal minimal differences between the treated and non-treated groups across several variables. This observation suggests that the treatment’s impact may be more nuanced, requiring a deeper exploration of underlying factors and conditions.

## Results

This paper examines the impact of ME on the racial and ethnic composition in nursing home admissions. Using the methodology developed by [6], alongside two-way fixed-effects (TWFE) for confirmation, the findings indicate significant policy effects: a decline in the proportion of Black residents and an increase for Hispanic and White residents. These results, shown in Table 1, maintain statistical significance even when controlling for additional variables, which slightly mitigates the impact but confirms the policy’s influence. The analysis, however, does not distinguish between private payers and those covered by Medicare or Medicaid, focusing solely on the racial and ethnic composition of nursing home residents. Additional insights from the [6] method, with various aggregation schemes, are presented in Table A5 in the Appendix, accounting for factors like calendar time and exposure length, alongside control variables and fixed effects.

**Table 1:** Medicaid expansion on nursing home residents race/ethnic composition

	TWFE	CS	
	(1)	(2)	(3)
(A) Aggregate Nursing Home Residents: Black			
Medicaid Expansion	-0.33^**^	-0.64^***^	-0.13^*^
	(0.11)	(0.13)	(0.06)
Adjusted $R^{2}$	0.96		
Observations	40400		
(B) Aggregate Nursing Home Residents: Hispanic			
Medicaid Expansion	0.24^**^	0.12^+^	0.38^*^
	(0.08)	(0.07)	(0.19)
Adjusted $R^{2}$	0.96		
Observations	38855		
(C) Aggregate Nursing Home Residents: White			
Medicaid Expansion	0.64^***^	0.81^***^	0.66^*^
	(0.19)	(0.20)	(0.28)
Adjusted $R^{2}$	0.92		
Observations	44862		
Controls Variables	Yes	No	Yes

Standard errors are in parentheses. Significance is denoted as follows: ^+^ $p<0.1$, ^*^ $p<0.05$, ^**^ $p<0.01$, ^***^ $p<0.001$. Two-way Fixed Effects (TWFE) and the Callaway and Sant’Anna (CS) results are included. Control variables include income per capita, population, occupancy rate, N.H. concentration, White and Black male population, political preference, and For-Profit facilities. Control group: Never treated. The results include year and county-fixed effects

The DiD analysis hinges on the parallel trends assumption, validated by the event-study estimates for Black and White residents, confirming consistent pre-policy trends and significant post-policy effects, as shown in Figure A4. However, the trend for Hispanic residents is less definitive, leading to inconclusive results for this group post-expansion. Complementary analyses, including a focused 2014 study and a shortened pre-expansion period study from 2006 to 2019. Focusing only on 2014 allows us to capture the initial effects of the ME. The unconditional parallel trends are provided in Figure A5. Including only 2014 is critical in understanding how quickly the policy change began its influence on nursing home admissions. Table A6 and Figure A5 show the results with shorter periods from 2006 to 2019. A shorter period more accurately reflects the healthcare landscape immediately preceding the expansion, thereby ensuring that the data is more directly applicable to the conditions and challenges the ME intended to address. Furthermore, this adjustment reduces the influence of unrelated historical events and economic fluctuations that occurred in the early 2000s, which could otherwise introduce noise and confound the analysis. These results reinforce the main findings, highlighting the immediate effects of the policy and enhancing the accuracy of the results.

To obtain deeper insights from the results, states are categorized by poverty rate and income inequality. This stratification helps determine how these factors influence nursing home admissions and the resulting disparities. States with greater inequality and poverty may have fewer resources for nursing homes, potentially heightening access barriers for low-income, minority groups. Moreover, states with progressive policies may approach nursing home admissions and racial equity differently. Categorizing states into high/low inequality and poverty groups reveals a more detailed picture of the drivers behind racial variations in nursing home admissions.

States were classified into high and low categories using their average poverty rates or Gini coefficients from 2000 to 2019. We calculated each state’s mean value over this period and then determined a national average as a benchmark. States with mean values above this national average were classified as high poverty or inequality, while those below were classified as low poverty or inequality. The definition of poverty follows the U.S. Census Bureau and is uniformly applied across states and periods. By using this standardized measure, the study maintains comparability and consistency in classifying states by poverty rates^1^.

Table 2 categorizes expansion and non-expansion states by their poverty and income inequality levels, using the [6] simple aggregation scheme and two-way fixed effects to validate findings. The ME’s intent to assist economically disadvantaged individuals allows for analysis of its impact in states with diverse economic profiles. States vary in Medicaid eligibility and economic disparities, which could affect the demographic makeup of nursing home residents and shed light on observed impacts from the ME. These economic factors at the state level are critical for understanding how the expansion might influence nursing home demographics.

**Table 2:** Medicaid expansion by poverty rate and income inequality

	Poverty rate				Income inequality			
	(1) TWFE		(2) CS		(3) TWFE		(4) CS	
	Low	High	Low	High	Low	High	Low	High
(A) Aggregate Nursing Home Residents: Black								
Medicaid Expansion	0.09	-0.72^***^	0.00	-0.49^**^	-0.27^*^	-0.43^*^	-0.10	-0.37^*^
	(0.08)	(0.20)	(0.09)	(0.17)	(0.12)	(0.19)	(0.08)	(0.14)
Adjusted $R^{2}$	0.96	0.95	22341	19343	0.97	0.95	22341	17469
Observations	20941	19639			22791	17789		
(B) Aggregate Nursing Home Residents: White								
Medicaid Expansion	0.42^+^	1.00^**^	0.67^*^	0.52	0.59^*^	0.62^+^	0.44	0.49^+^
	(0.25)	(0.33)	(0.30)	(0.33)	(0.24)	(0.34)	(0.31)	(0.30)
Adjusted $R^{2}$	0.84	0.92	23461	21519	0.91	0.91	25518	19462
Observations	23500	21542			25544	19498		

Standard errors are in parentheses. Significance is denoted: ^+^ $p<0.1$, ^*^ $p<0.05$, ^**^ $p<0.01$, ^***^ $p<0.001$. Two-way Fixed Effects (TWFE) and the Callaway and Sant’Anna (CS) results are included. Control variables include income per capita, population, occupancy rate, N.H. concentration, White and Black male population, political preference, and For-Profit facilities. The results include year and county-fixed effects. The classification of states by poverty rate and income inequality is detailed in Results section

In this study, I delve into how ME influences nursing home demographics differently across states divided by poverty and income inequality. The results are presented in Table 2. For states with low poverty rates, ME doesn’t show a significant impact on Black residents. However, in states with high poverty rates, ME significantly decreases the admissions of Black residents, hinting at how the policy’s effects can vary with the underlying economic conditions. This decrease might be due to nursing homes in high-poverty states adjusting their admissions preferences in anticipation of more Medicaid enrollees post-ME, possibly favoring private-pay residents.

In the context of states with low poverty rates, ME shows a slight positive effect on the admissions of White residents, indicating that even in areas with generally lower Medicaid dependency, ME can positively influence admissions among White populations. This could imply less racial diversity in these states, as suggested by Figure A6, which shows lower poverty rates correlating with higher White populations and higher poverty with larger Black populations. It also might suggest that ME’s benefits are not confined to high-poverty areas but extend across different economic backgrounds. Conversely, in high-poverty states, the expected increase in admissions for White residents following ME does not manifest as significantly. This could be indicative of a complex interplay between Medicaid expansion and the specific socioeconomic fabric of these states, where increased Medicaid enrollment does not necessarily translate into higher admissions for White residents, possibly due to a saturated Medicaid market or a shift in nursing homes’ preferences towards privately insured individuals.

Shifting the lens to income inequality, the pattern becomes even more interesting. In high-income inequality states, ME leads to a noticeable decrease in Black resident admissions and a slight increase in White resident admissions, reflecting how disparities in economic status within states might influence the policy’s outcomes. These findings underscore the importance of considering state-level poverty and income inequality when analyzing the effects of ME on nursing home demographics. They open up questions about the role of economic diversity and healthcare access disparities, setting the stage for a deeper dive into the mechanisms behind these patterns, particularly nursing homes’ payment preferences.

### Mechanisms

To explain the observed decrease in Black residents and increase in White residents post-ME, this study considers the influence of nursing home admission preferences and financial incentives. As mentioned earlier, the alternative hypothesis posits that nursing homes may prefer residents with private insurance or more personal funds, potentially due to biases or economic incentives, as these residents might offer higher reimbursement rates. The research further investigates this by examining payment sources and bed availability, with findings detailed in Table 3.

**Table 3:** Medicaid expansion on nursing home residents: mechanisms

	(1)	(2)	(3)	(4)
	P. Medicaid	P. Medicare	P. Private	T. Beds
Medicaid Expansion	-0.42^+^	-0.06	0.48^*^	-9.44^**^
	(0.25)	(0.15)	(0.24)	(3.20)
Observations	45479	45479	45479	45479

Standard errors are in parentheses. Significance is denoted as follows: ^+^ $p<0.1$, ^*^ $p<0.05$, ^**^ $p<0.01$, ^***^ $p<0.001$. The variables P.Medicaid in column (1), P.Medicare in (2), P.Private in (3), and T.Beds in (4) were examined as dependent variables. Control variables include income per capita, population, occupancy rate, N.H. concentration, White and Black male population, political preference, and For-Profit facilities. Control group: Never treated. The results include year and county-fixed effects. Dependent variables are defined in Mechanisms section

The data regarding forms of payment can be divided into “P.Medicaid” and “P.Medicare”. These variables show the proportion of residents whose primary support is either Medicaid or Medicare. In Figure A7, it can be appreciated the correlation between population by race/ethnicity and the shares of either primary support. This graph shows that shares of Medicaid-supported patients are highly affected by race/ethnicity. This is an important background for the results of Table 3. Post-policy implementation findings indicate a statistically significant decrease in the proportion of Medicaid-supported residents in column 1 of Table 3. In contrast, in column 2, the Medicare-supported resident share remains largely unchanged, a predictable outcome considering that the ME does not directly target Medicare beneficiaries. This shift suggests two potential compensatory responses by nursing homes: an increase in privately funded residents or a reduction in total bed availability.

The variable for private-pay nursing home residents, “P.Private”, is calculated by subtracting the proportions of Medicaid and Medicare residents from the total. This is key for assessing if homes prefer privately funded residents. Column 3 of Table 3 shows a significant increase in private residents after ME, supporting the theory of shifting preferences. Additionally, column 4 shows a statistically significant drop in total nursing home beds post-expansion, “T.Beds”, suggesting a link between reduced Medicaid resident shares and facility capacity. However, the specific impact on beds designated for Medicaid remains unclear due to data limitations on post-expansion allocations. This leaves the relationship between the Medicaid-assigned beds and ME open for clarification in future research.

To shed light on the role of these mechanisms in explaining the main results, the main specification might be modified by adding each of them to control for their effects on racial/ethnic composition on nursing home admissions. These results are reported in Table 4 by including TWFE and CS estimators. The main reason for including TWFE in this table is due to the reporting of the covariates. It is not possible to obtain coefficients from covariates from the software package provided by [6] even when these covariates are included.

**Table 4:** Medicaid expansion on nursing home residents race/ethnic composition with mechanisms

	(1)		(2)		(3)	
	TWFE	CS	TWFE	CS	TWFE	CS
(A) Aggregate Nursing Home Residents: Black						
Medicaid Expansion	-0.31^**^	-0.13^+^	-0.32^**^	-0.15^*^	-0.33^**^	-0.16^*^
	(0.11)	(0.07)	(0.11)	(0.07)	(0.11)	(0.07)
P. Medicaid	0.02^***^					
	(0.00)					
P. Private			-0.01^**^			
			(0.00)			
T. Beds					-0.00	
					(0.00)	
Adjusted $R^{2}$	0.96		0.96		0.96	
Observations	40580		40580		40580	
(B) Aggregate Nursing Home Residents: White						
Medicaid Expansion	0.61^**^	0.41^*^	0.62^**^	0.45^*^	0.63^**^	0.48^*^
	(0.19)	(0.20)	(0.19)	(0.21)	(0.19)	(0.20)
P. Medicaid	-0.03^***^					
	(0.01)					
P. Private			0.03^***^			
			(0.01)			
T. Beds					0.00^*^	
					(0.00)	
Adjusted $R^{2}$	0.92		0.92		0.92	
Observations	45042		45042		45042	

Standard errors are in parentheses. Significance is denoted as follows: ^+^ $p<0.1$, ^*^ $p<0.05$, ^**^ $p<0.01$, ^***^ $p<0.001$. Two-way Fixed Effects (TWFE) and the Callaway and Sant’Anna (CS) results are included. Control variables include income per capita, population, occupancy rate, N.H. concentration, White and Black male population, political preference and For-Profit facilities. Additional variables are included: in columns (1) P.Medicaid, (2) P.Private, and (3) T.Beds. These variables are defined in Mechanisms section. Control group: Never treated. The results include year and county-fixed effects

Table 4 shows that, for Black residents, ME shows a negative association with admissions, suggesting a decrease post-expansion, despite a positive association of Medicaid coverage with increased admissions for this group. This apparent contradiction indicates that while Medicaid coverage at an individual level facilitates access to nursing homes for Black residents, the broader policy implications of ME might lead to a net decrease in admissions, possibly due to increased competition or systemic preferences within nursing homes. Conversely, for White residents, ME is positively associated with increased admissions, but higher Medicaid coverage correlates with a decrease, hinting at a preference for other forms of payment such as private insurance.

Furthermore, column 3 of Table 4, which incorporates the total number of beds available, reveals a significant effect exclusively for White residents. An increase in the total beds available is linked to a rise in admissions for White residents, with no corresponding impact on Black admissions. This differential effect underscores the complex interplay between individual coverage benefits and the broader implications of ME, which appear to diverge along racial lines. It hints at systemic biases in nursing home admissions post-ME, necessitating a more detailed exploration of the underlying mechanisms, such as payment preferences and racial dynamics.

The preference for private-pay residents in the aftermath of ME, as inferred from the increase in admissions for White residents, suggests that the policy may inadvertently catalyze a shift towards more private-pay beds, effectively sidelining Medicaid-reliant residents. This shift predominantly benefits White residents, who generally have a lower share of Medicaid coverage. The observed increase in private-pay residents post-expansion, thus, points to White residents as the primary beneficiaries of nursing home admissions following ME.

#### Classification by poverty rate

Investigating the mechanisms through which ME operates across states with different poverty levels offers further insights into its broader impacts. After initially segmenting states by poverty rates, we now turn our attention to the specific effects of ME on key mechanisms such as Medicaid, Medicare, private pay shares, and the total number of nursing home beds within these groupings. This detailed exploration is presented in Table A8 in the Appendix.

For Medicaid-supported residents, shown in panel A, the impact is predominantly observed only in low-poverty states. This counterintuitive finding suggests that in states with lower poverty levels, where economic conditions might favor a larger proportion of the population being able to afford private healthcare options, ME may not lead to the expected increase in Medicaid-supported nursing home admissions. This could be due to a variety of factors, including the availability of alternative care options, demographic shifts, or a stronger preference for private payment methods in these regions. States with lower levels of poverty may have a higher proportion of residents who prefer or have the means to opt for privately paid care, leading to a nuanced impact of ME on the payer mix in nursing homes. This preference for private payment could influence the composition of nursing home admissions, potentially skewing it away from Medicaid-supported residents despite the expansion intended to increase their access. Private pay shares, in panel C, show an increase in low-poverty states following ME. This could also indicate a shift towards a larger proportion of privately paying residents in these states, possibly due to a combination of ME’s effects on the payer mix and the existing socio-economic landscape favoring private insurance over Medicaid.

Regarding Medicare residents, in panel B, as anticipated, there is no significant change post-ME across both high and low-poverty states. This is expected since ME targets Medicaid, not Medicare beneficiaries. The total number of beds in nursing homes, as shown in panel D, presents a significant decrease in high-poverty states, which may highlight resource constraints exacerbated by ME, affecting the availability of spaces for potential Medicaid beneficiaries. The contrasting effects of ME in high and low-poverty states reveal the nuanced ways in which state-level socio-economic factors interact with policy changes. In low-poverty states, the shift towards private payers and the reduction in Medicaid-supported residents post-ME suggest that economic disparities and the structure of healthcare financing play crucial roles in shaping the outcomes of Medicaid expansion. Conversely, in high-poverty states, the decrease in available nursing home beds post-expansion underscores the challenges of addressing care needs in areas with higher poverty rates.

The study delves deeper into the mechanisms at play, particularly focusing on Medicaid, Medicare, and private-pay shares, as well as the total number of nursing home beds. These findings are detailed in Table A9 in the Appendix. For Black nursing home residents in panel (A) of Table A9, the data delineates a clear dichotomy between low- and high-poverty states. In low-poverty states, ME does not significantly impact Black admissions. Contrastingly, in high-poverty states, ME markedly decreases Black admissions, pointing to a nuanced interaction where ME’s intent to increase access may tighten admission rates for Black residents, possibly through an increased competition for limited spaces or a shift towards private pay preferences by nursing homes. White nursing home residents’ admissions only in low-poverty states see a positive and significant effect from ME.

In analyzing the effects of ME on nursing home admissions, distinct outcomes for Black and White residents across states with varying poverty levels can be observed. An important aspect of this analysis involves the examination of forms of payment as a mechanism to understand these impacts further. The significant positive association between P.Medicaid and admissions for Black residents in high-poverty states underscores a complex scenario. While ME aims to expand healthcare access, this association suggests that individual Medicaid coverage could facilitate access to nursing homes for Black residents, echoing the policy’s intention. However, the overall negative impact of ME in high-poverty states indicates that, despite increased individual access to Medicaid, systemic factors or nursing home preferences might lead to a net decrease in admissions.

Conversely, the relationship between P.Medicaid and White nursing home residents presents a slight negative correlation in low-poverty states, suggesting that an increase in Medicaid coverage does not similarly boost admissions for White residents. This could reflect a preference shift within nursing homes towards privately insured individuals, possibly influenced by ME. Notably, the positive impact of ME on White residents in low-poverty states, although moderated, remains significant, indicating that White residents might benefit more from ME in areas with less economic disparity. These findings show the need for a deeper exploration of the mechanisms at play, particularly the interaction between race/ethnicity, forms of payment, and ME. The apparent preference for privately paying residents, coupled with the nuanced effects of Medicaid coverage, underscores the complexity of ME’s impact on nursing home admissions.

Differences in state poverty levels and their demographic compositions play a critical role in shaping the observed impacts of ME on nursing home admissions. States with lower poverty rates often have higher proportions of White populations and potentially greater access to private healthcare options. This demographic and economic context may lead to a more pronounced positive effect of ME on White nursing home admissions, as seen in low-poverty states in Table A9, where the expansion could indirectly benefit individuals with access to multiple care options. Conversely, states with higher poverty rates tend to exhibit greater racial diversity, including larger Black and Hispanic populations who may rely more heavily on Medicaid due to socioeconomic constraints. In these high-poverty, more diverse states, the competition for limited Medicaid-supported nursing home slots may intensify following ME, potentially leading to the decrease in admissions for Black residents seen in Table A9. This interplay suggests that state-level economic and demographic characteristics, alongside nursing homes’ payment preferences, significantly influence how ME’s effects manifest across different racial and ethnic groups.

#### Forms of Payment and Race/Ethnicity Interaction

Additional results are obtained by examining the effects of the interaction between racial groups and payment types. The construction of interaction terms is important for exploring the nuanced impact of ME on nursing home admissions across different racial groups and payment methods. The interaction terms are formulated by multiplying the county-level share of Medicaid or private insurance coverage by the aggregate number of nursing home admissions for Black and White residents within each county. This approach serves two primary purposes: First, by constructing these interaction terms, I aim to distinguish the specific influence of Medicaid and private insurance coverage on nursing home admissions for each racial group. This distinction is critical for understanding whether payment method preferences or racial disparities predominantly drive the observed patterns in nursing home admissions following ME. Second, the interaction terms enable a granular analysis of ME’s effects, revealing how the policy may differentially impact nursing home admissions based on both the residents’ racial background and their primary payment method. This analysis is important for identifying unintended consequences of ME, such as reinforcing existing disparities or altering the composition of nursing home populations in ways that may not align with policy goals.

Table 5 reveals statistically significant effects of ME on the interaction between Medicaid and Black residents called Medicaid-Black and between private pay and White residents called Private-White. Specifically, ME is associated with a reduction in Medicaid-reliant Black residents and an increase in Private-Paying White residents in nursing homes. These findings confirm that ME’s implementation may have inadvertently contributed to a shift in the racial and payment composition of nursing home admissions, highlighting a potential payment-based preference within nursing homes that affects racial groups differently. This table also shows non-significant effects of the policy implementation on White residents with Medicaid support. The lack of significant impact on Medicaid-White residents could suggest that the potential access to care disparities are not merely a result of Medicaid coverage but also involve other factors, such as nursing home admission policies. Building on these insights, I then incorporate these mechanisms into the main regression models to assess their influence on the overall effect of ME on nursing home admissions by race. Table 6 includes the Medicaid-Black and Private-White interactions within the broader model assessing the impact of ME on Black and White nursing home residents.

**Table 5:** Medicaid expansion effects on interaction terms

	(1)	(2)	(3)	(4)
	Medicaid-White	Medicaid-Black	Private-White	Private-Black
Medicaid Expansion	6.54	-13.16^*^	56.71^*^	-2.50
	(29.43)	(5.64)	(23.16)	(2.65)
Observations	45007	40545	45007	40545

Standard errors are in parentheses. Significance is denoted: ^+^ $p<0.1$, ^*^ $p<0.05$, ^**^ $p<0.01$, ^***^ $p<0.001$. Two-way Fixed Effects (TWFE) and the Callaway and Sant’Anna (CS) results are included. Control variables include income per capita, population, occupancy rate, N.H. concentration, White and Black male population, political preference, and For-Profit facilities. The Dependent variables: Private-Black and Medicaid-Black refer to Black residents who pay privately or via Medicaid. Similarly, Private-White and Medicaid-White refer to White residents who pay privately or via Medicaid. The results include year and county-fixed effects

**Table 6:** Medicaid expansion on nursing home residents composition with interaction terms

	(1)		(2)	
	TWFE	CS	TWFE	CS
(A) Aggregate N.H. Residents: Black				
Medicaid Expansion	0.12^*^	-0.20^**^	-0.29^**^	-0.15^*^
	(0.06)	(0.07)	(0.11)	(0.07)
Medicaid-Black	0.01^***^			
	(0.00)			
Private-White			-0.00^***^	
			(0.00)	
Adjusted $R^{2}$	0.99		0.96	
Observations	40545		40456	
(B) Aggregate N.H. Residents: White				
Medicaid Expansion	0.20	0.37^+^	0.51^**^	0.41^*^
	(0.21)	(0.22)	(0.19)	(0.20)
Medicaid-Black	-0.01^***^			
	(0.00)			
Private-White			0.00^***^	
			(0.00)	
Adjusted $R^{2}$	0.94		0.92	
Observations	40456		45007	

Standard errors are in parentheses. Significance is denoted: ^+^ $p<0.1$, ^*^ $p<0.05$, ^**^ $p<0.01$, ^***^ $p<0.001$. Two-way Fixed Effects (TWFE) and the Callaway and Sant’Anna (CS) results are included. Control variables include income per capita, population, occupancy rate, N.H. concentration, White and Black male population, political preference, and For-Profit facilities. Additional interaction variables: Medicaid-Black refers to Black residents who pay via Medicaid and Private-White to White residents who pay privately. The results include year and county-fixed effects

For Black residents, the inclusion of interaction terms offers a detailed view of ME’s impact, particularly on those reliant on Medicaid. While the coefficient for Medicaid Expansion indicates a reduction in the admissions of Black residents, this effect becomes more pronounced upon incorporating the Medicaid-Black interaction term. This observation suggests that ME, contrary to its intention to broaden access, specifically diminishes admissions for Medicaid-reliant Black residents. Such a reduction in admissions contributes to a more substantial decrease compared to the initial estimates presented in Table 1. The analysis further elaborated in Table 5, highlights a key dynamic: while an increase in Medicaid-supported Black residents theoretically should boost overall admissions, ME’s direct impact-reducing Medicaid-reliant Black admissions-counteracts this potential. Consequently, the overall effect of ME on Black admissions becomes markedly more negative with the interaction term accounted for. Additionally, it can be observed that an increase in privately paying White residents, decreases the aggregate number of Black residents in nursing homes.

In the analysis of White residents, integrating the Private-White interaction term into the main regression, as shown in column 2 of Table 6, not only moderates the effect of ME on White residents but also provides insight into the mechanism behind this change. This attenuation, moving from the initial coefficient detailed in Table 1, suggests that ME notably facilitates an increase in admissions among White residents with private insurance. This specific enhancement of admissions for privately insured White residents under ME suggests that ME’s broader access initiatives might be differentially benefiting residents based on their insurance type, with private insurance holders, particularly White residents, finding more favor in the admissions process. This nuanced finding becomes even more compelling when considering the Medicaid-Black interaction, presented in column 1. The inclusion of this term further adjusts the ME effect on White admissions downward, suggesting that the expansion’s overall impact is modulated by the racial composition of Medicaid-reliant admissions. The attenuation of ME’s effect means that while ME aims to increase healthcare access, the actual realization of this goal is skewed by existing payment and racial dynamics within nursing home admissions.

## Discussion

The study examines how preferences and cost concerns might drive the post-ME admission changes in nursing homes. It considers that increased costs could discourage minority admissions, possibly due to varying Medicaid reimbursements or local cost differences. Nursing homes might prefer White residents, anticipating better financial returns from private insurance or resources. This trend may not reflect racial prejudice alone but economic strategy, with homes possibly perceiving care for minority groups as costlier and more resource-intensive [37]. Such economic motives may intersect with implicit biases, thus influencing admission patterns and highlighting the complex dynamics at play in nursing home decisions.

While preferences and economic factors elucidate the rise in White nursing home admissions, the decline in minority admissions is less clear. Various theories could illuminate this, such as improved healthcare access for low-income individuals, including minorities, as a result of ME [22]. This enhanced access may lessen reliance on nursing home care, thereby reducing admission rates among these groups. Additionally, if the quality of healthcare for low-income populations has risen, the need for such care among minorities may decline, though the impact on healthcare quality varies [23].

The expansion might also affect choices between formal healthcare and informal caregiving [38]. Better healthcare access could decrease minority populations’ dependence on informal caregivers. Quality differences in care types matter too-if minorities have better informal care, they may be less likely to choose nursing homes, whereas White residents might favor formal care due to lesser quality informal care options [39]. Cultural and language barriers could further complicate minorities’ use of formal care systems. Despite Medicaid Expansion facilitating financial access, racial and ethnic minorities, particularly those with limited English proficiency, encounter significant barriers to care [40, 41]. Implementing online translation tools could serve as steps toward mitigating these challenges and improving healthcare outcomes [42]. Cost is a significant consideration; if informal care is more accessible for minorities [43], it may be preferred over more expensive nursing homes. Conversely, White residents might turn to formal care if affordable informal care is scarce [44]. Finally, historical discrimination in healthcare may affect trust levels; minorities with lower trust may opt for informal care, while higher trust among White populations could lead to more nursing home utilization [45].

Despite the insights provided by this study, several limitations warrant mention. First, the use of county-level panel data, while extensive, may mask individual-level variations in nursing home admissions and experiences. Individual data could offer more nuanced insights into the decision-making processes of the nursing homes and the families or individuals seeking care. The potential impact of informal care on nursing home admissions is another area that remains unexplored due to data limitations, suggesting an important direction for future research. Second, our analysis is constrained by the available data on payment methods and does not fully explore the financial dynamics between Medicaid and private payers. This limitation restricts our ability to comprehensively understand how economic factors influence nursing home admissions across different racial groups. Future research should aim to address these limitations, exploring the multifaceted impacts of healthcare policies on diverse populations. Lastly, the analysis does not account for the quality of care or residents’ outcomes in nursing homes, which are critical aspects of evaluating the ME’s success. Even though the findings of [28] suggest clear differences in the quality of care received by different races, this study does not directly address these disparities due to its focus on admissions data.

## Conclusions

The Affordable Care Act (ACA), enacted in 2010 is an important milestone for improving the health care coverage of American citizens. This paper analyzes the ME-a key ACA provision-and its impact on the racial and ethnic composition of nursing home admissions. The study is driven by increasing concerns over racial and economic inequality, especially given the disproportionate poverty among Black and Hispanic communities, and the challenges in long-term care affordability and access. It hypothesizes that ME should lead to an increase in nursing home admissions among these economically disadvantaged minorities.

This study employs the [6] difference-in-differences estimation method on U.S. county-level panel data from 2000 to 2019. Findings indicate that the ME has not diversified nursing home demographics but rather has led to a more uniform racial composition, with fewer Black residents and an increase in White and Hispanic residents. However, the data for Hispanic residents are less definitive, possibly due to lower statistical significance and challenges meeting parallel trend assumptions, echoing broader issues identified in the literature such as language and cultural barriers in healthcare access [46].

The findings indicate that nursing home preferences and cost strategies may increasingly favor White residents, likely due to their higher likelihood of having private insurance. This bias towards privately paying residents within nursing homes contributes to the observed decrease in admissions for Black residents and an increase for White residents following Medicaid Expansion. This shift underscores a potential payment preference within nursing homes, directly influencing the racial composition of admissions and highlighting the complex dynamics between policy implementation and healthcare access across different racial groups.

### Supplementary Information

Supplementary Material 1.

## Data Availability

No datasets were generated or analysed during the current study.

## Abbreviations

ACA: Affordable care act
ATT: Average treatment effect on the treated
CS: Callaway and Sant’Anna
DiD: Difference-in-differences
ME: Medicaid expansion
NH: Nursing home
TWFE: Two-way fixed effects

## Footnotes

1 The state’s classification is shown in the Appendix in Table A7 for poverty rate and for income inequality.
