# Supplementary material for: The effects of Medicaid expansion on the racial/ethnic composition within nursing home residents
Source: Health Econ Rev. 2024 Jun 20;14:43. doi: 10.1186/s13561-024-00517-3 (PMC11191276; doi:10.1186/s13561-024-00517-3)
Supplement: Supplementary file 1 — Supplementary Material 1. [file 13561_2024_517_MOESM1_ESM.pdf]

Electronic Supplementary Material

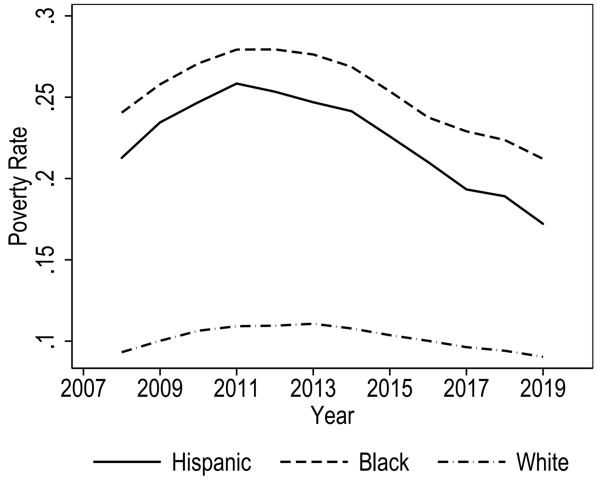

Figure A1: Poverty Rate by Race/Ethnicity

Note: This figure plots the poverty rates by race/ethnicity in the U.S. from 2008 to 2019. Source: KFF estimates based on the American Community Survey.

Table A1: Year of Implementation of the Affordable Care Act

State	Year	State	Year	State	Year
Alaska	2015	Michigan	2014	Virginia	2019
Arizona	2014	Minnesota	2014	Washington	2014
Arkansas	2014	Missouri	2021	West Virginia	2014
California	2014	Montana	2016	Alabama	N.I
Colorado	2014	Nebraska	2020	Florida	N.I
Connecticut	2014	Nevada	2014	Georgia	N.I
Delaware	2014	New Hampshire	2014	Kansas	N.I
District of Columbia	2014	New Jersey	2014	Mississippi	N.I
Hawaii	2014	New Mexico	2014	North Carolina	N.I
Idaho	2020	New York	2014	South Carolina	N.I
Illinois	2014	North Dakota	2014	South Dakota	N.I
Indiana	2015	Ohio	2014	Tennessee	N.I
Iowa	2014	Oklahoma	2021	Texas	N.I
Kentucky	2014	Oregon	2014	Wisconsin	N.I
Louisiana	2016	Pennsylvania	2015	Wyoming	N.I
Maine	2018	Rhode Island	2014		
Maryland	2014	Utah	2020		
Massachusetts	2014	Vermont	2014		

Note: This table reports the year of implementation of the Affordable Care Act by state. District of Columbia and Alaska are not included in the main data. Additionally, states which implemented the expansion after 2019 are considered as ”never treated”.

Table A2: Correlation Matrix

	(1)	(2)	(3)	(4)	(5)	(6)	(7)	(8)	(9)	(10)	(11)	(12)	(13)
Agg. Black	1												
Agg. Hispanic	-0.0745^{***}	1											
Agg. White	-0.768^{***}	-0.491^{***}	1										
Log Income p.c.	-0.0873^{***}	-0.0208^{***}	0.0539^{***}	1									
Log Population	0.130^{***}	0.0895^{***}	-0.168^{***}	0.258^{***}	1								
Occupancy	0.0717^{***}	-0.123^{***}	0.0181^{***}	-0.0336^{***}	0.130^{***}	1							
N.H. Concentration	0.0360^{***}	0.00407	-0.0335^{***}	-0.253^{***}	-0.704^{***}	-0.0125^{**}	1						
Log White Male Pop.	-0.0413^{***}	0.0918^{***}	-0.0202^{***}	0.265^{***}	0.984^{***}	0.130^{***}	-0.706^{***}	1					
For-Profit Facility	0.182^{***}	0.0889^{***}	-0.160^{***}	-0.108^{***}	0.216^{***}	-0.165^{***}	-0.126^{***}	0.179^{***}	1				
P. Medicaid	0.384^{***}	0.0812^{***}	-0.349^{***}	-0.417^{***}	-0.0358^{***}	0.0721^{***}	0.219^{***}	-0.120^{***}	0.201^{***}	1			
P. Medicare	0.0737^{***}	0.0362^{***}	-0.0573^{***}	0.168^{***}	0.441^{***}	0.000339	-0.250^{***}	0.419^{***}	0.305^{***}	-0.202^{***}	1		
Intensity Care	0.102^{***}	0.0579^{***}	-0.0853^{***}	0.530^{***}	0.153^{***}	-0.0413^{***}	-0.0310^{***}	0.119^{***}	0.0831^{***}	-0.0548^{***}	0.275^{***}	1	
T. Beds	0.0831^{***}	0.0704^{***}	-0.132^{***}	0.229^{***}	0.569^{***}	0.0544^{***}	-0.373^{***}	0.554^{***}	0.0560^{***}	-0.0490^{***}	0.132^{***}	0.0405^{***}	1

Note: In the correlation matrix significance is denoted as $^{+}$ $p < 0.1$, * $p < 0.05$, ** $p < 0.01$, *** $p < 0.001$, and the variables are as follows: (1) Aggregate Black Population, (2) Aggregate Hispanic Population, (3) Aggregate White Population, (4) Log Income p.c., (5) Log Population, (6) Occupancy, (7) N.H. Concentration, (8) Log White Male Pop., (9) For-Profit Facility, (10) P.Medicaid, (11) P.Medicare, (12) Intensity Care, (13) T.Beds. These variables are defined in section 3.1.

Table A3: Summary Statistics

	Observations	Mean	Min	Max	St.Dev.
Agg. Black	50088	8.40	0	100	13.64
Agg. Hispanic	47595	2.55	0	100	9.26
Agg. White	56734	87.46	0	100	15.97
Log Income p.c.	57381	10.39	9.24	12.35	0.29
Log Population	57381	10.40	6.04	16.13	1.37
Occupancy	48856	81.91	1.67	100	12.34
N.H. Concentration	48860	0.50	0	1	0.33
Log White Male Pop.	54014	9.59	5.38	15.10	1.32
Log Black Male Pop.	53807	6.26	0	13.38	2.45
For-Profit Facility	48863	62.94	0	100	36.06
Political Preference	57340	1.79	1	2	0.41
P.Medicaid	48864	65.92	0	100	12.74
P.Medicare	48864	10.70	0	100	6.52
T.Beds	48864	580.55	8	40916	1495.72

Note: The construction of this data set and the definitions of the variables are discussed in section 2.

Table A4: Covariate's Summary Statistics by Treated Group

	Mean	St.Dev.	Min	Max
Log Income p.c.				
Treated	10.42	0.2	9.53	12.19
Non-Treated	10.35	0.28	9.23	12.34
Log Population				
Treated	10.61	1.38	6.87	16.12
Non-Treated	10.17	1.32	6.04	15.36
Occupancy				
Treated	83.22	11.18	4.44	100
Non-Treated	80.51	13.30	1.67	100
N.H. Concentration				
Treated	0.45	.33	0	1
Non-Treated	0.54	.33	0.01	1
Log White Male Pop.				
Treated	9.85	1.32	6.14	15.09
Non-Treated	9.29	1.26	5.37	14.31
Log Black Male Pop.				
Treated	6.21	2.41	0	13.38
Non-Treated	6.32	2.49	0	12.99
Political Preference				
Treated	1.73	.44	1	2
Non-Treated	1.85	0.35	1	2
For-Profit Facility				
Treated	60.29	34.52	0	100
Non-Treated	65.74	37.41	0	100

Note: The construction of this data set and the definitions of the variables are discussed in section 2.

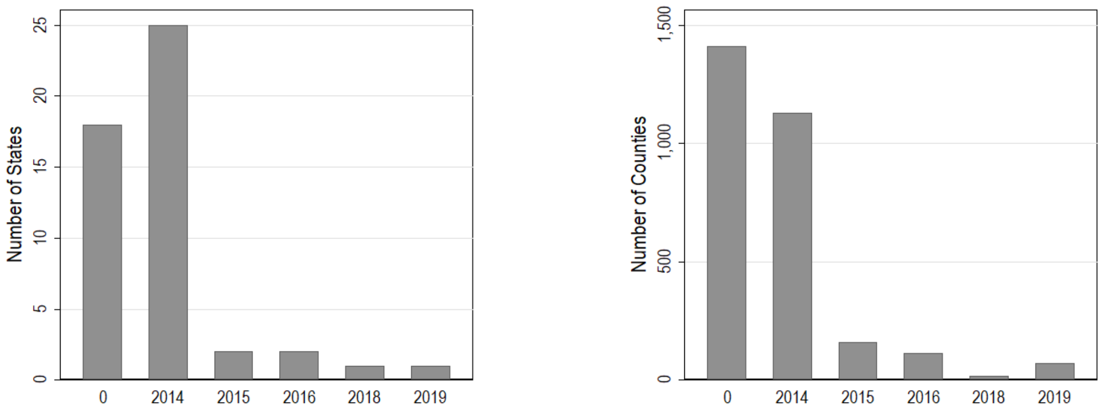

(a) States (b) Counties

Figure A2: Number of States/Counties Implementing the ACA by Year

Note: This graph shows the number of states in panel (a) and counties in panel (b) that implemented the ACA by year of implementation. Data sources are detailed in section 2.

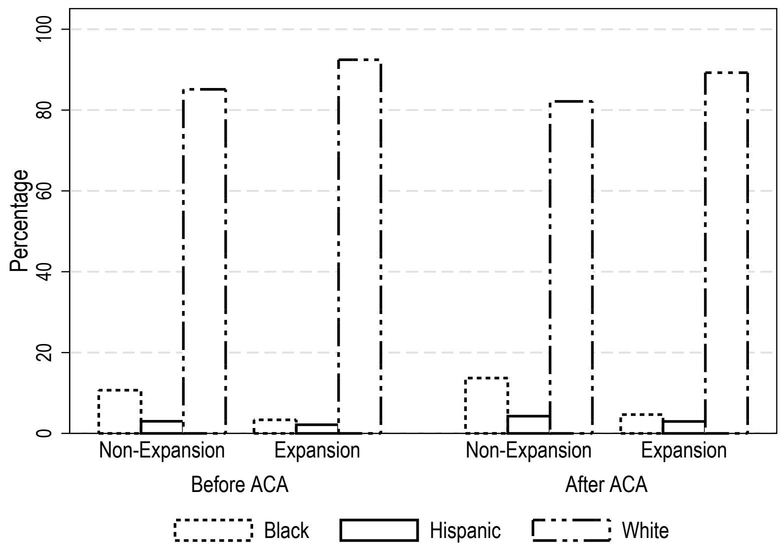

Figure A3: Race/Ethnicity Composition in Nursing Homes around Medicaid Expansion

Note: This figure classifies the composition of nursing home facilities between expansion and non-expansion states by race/ethnicity before and after the Medicaid expansion. The data used is discussed in section 2.

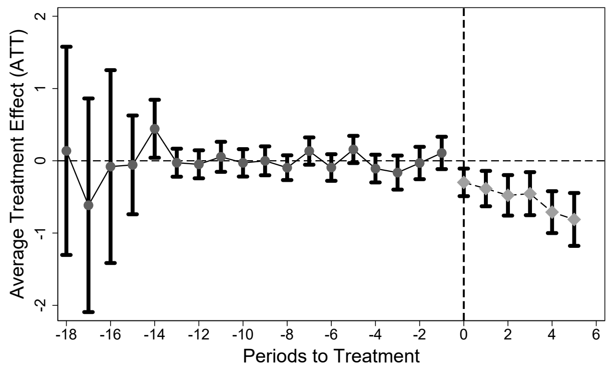

(a) Agg. Black Residents

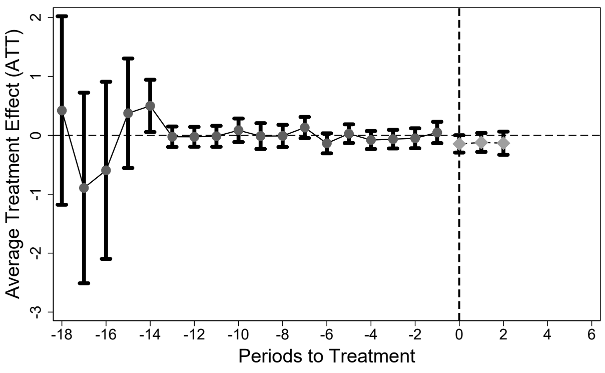

(b) Agg. Black Residents with Control Var.

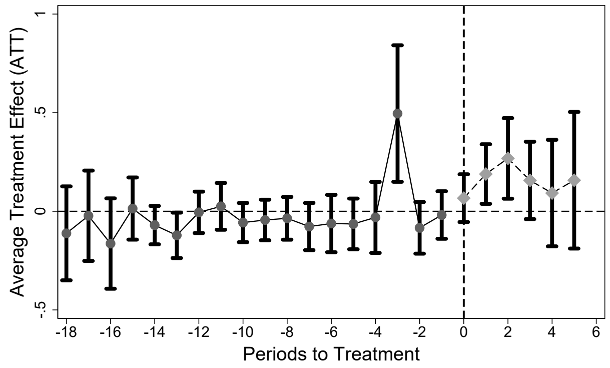

(c) Agg. Hispanic Residents

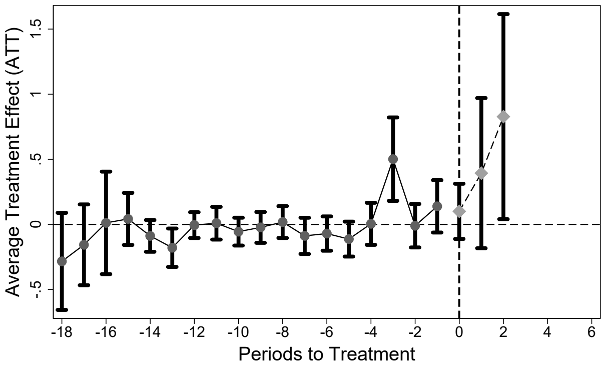

(d) Agg. Hispanic Residents with Control Var.

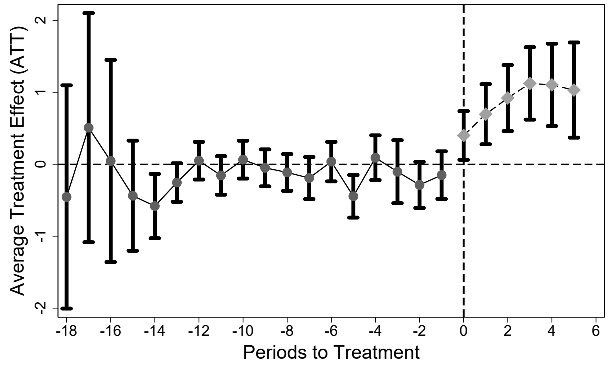

(e) Agg. White Residents

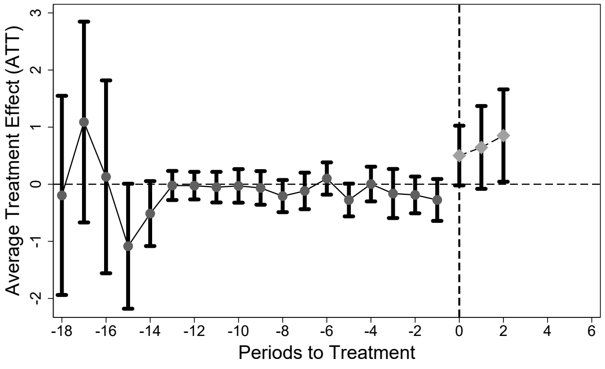

(f) Agg. White Residents with Control Var.

Figure A4: Medicaid Expansion on Nursing Home Residents Race/Ethnicity Composition

Note: The graphs plot the estimates and 95% confidence intervals for the unconditional ((a), (c), (e)) and conditional ((b), (d), (f)) parallel trends for the average effect of the Medicaid expansion on Black, Hispanic, and White nursing home residents. These results are based on the 'never-treated' group from the year 2000 to 2019.

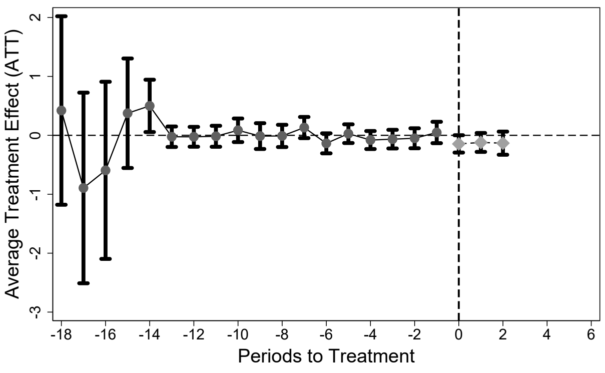

(a) Agg. Black Residents (2014)

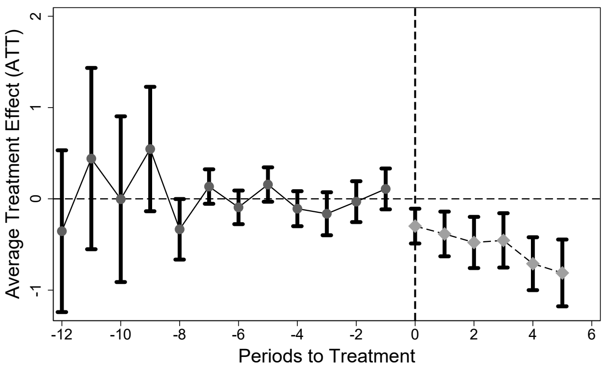

(b) Agg. Black Residents (2006-2019)

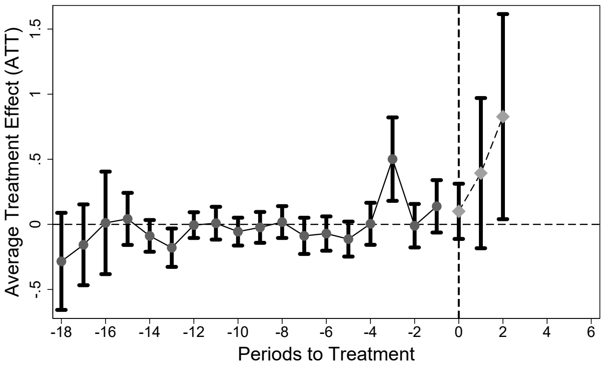

(c) Agg. Hispanic Residents (2014)

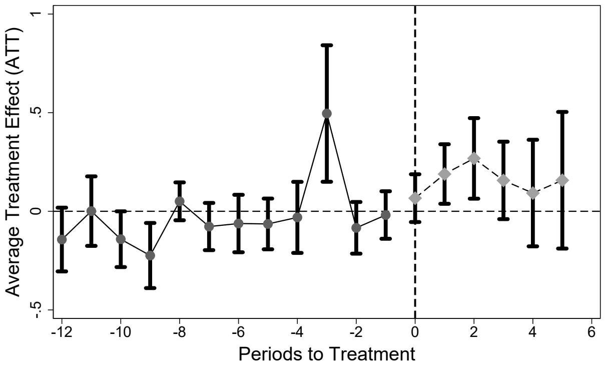

(d) Agg. Hispanic Residents (2006-2019)

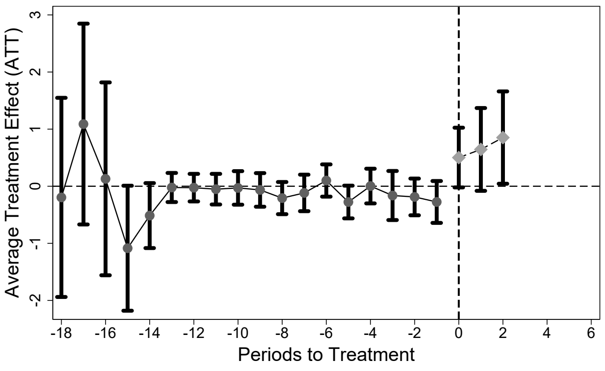

(e) Agg. White Residents (2014)

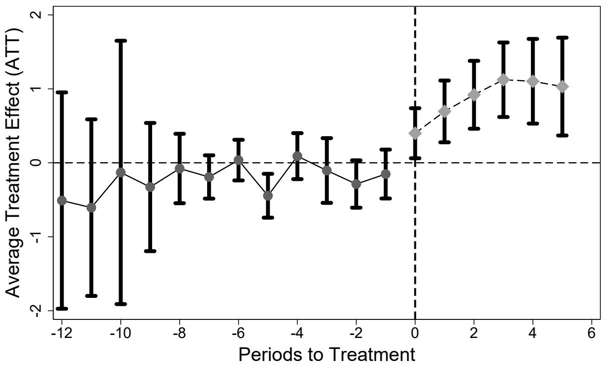

(f) Agg. White Residents (2006-2019)

Figure A5: Medicaid Expansion on Nursing Home Residents Race/Ethnicity Composition

Note: The graphs plot the estimates and 95% confidence intervals for the unconditional parallel trends for the average effect of the Medicaid expansion on Black, Hispanic, and White nursing home residents. These results are based on the 'never-treated' group. Panels ((a), (c), (e)) report results for the 2014 group, and panels ((b), (d), (f)) for the years 2006-2019.

Table A5: Additional CS Aggregation Schemes

	(1) Simple	(2) Calendar	(3) Group	(4) Length
(A) Aggregate Nursing Home Residents: Black				
Medicaid Expansion	-0.13*	-0.13*	-0.15^{+}	-0.13*
	(0.06)	(0.06)	(0.08)	(0.07)
Observations	40400	40400	40400	40400
(B) Aggregate Nursing Home Residents: Hispanic				
Medicaid Expansion	0.38*	0.37*	0.37*	0.41*
	(0.19)	(0.18)	(0.18)	(0.20)
Observations	38855	38855	38855	38855
(C) Aggregate Nursing Home Residents: White				
Medicaid Expansion	0.66*	0.65*	0.60*	0.67*
	(0.28)	(0.28)	(0.26)	(0.29)
Observations	44862	44862	44862	44862

Note: Standard errors are in parentheses. Significance is denoted as follows: $^{+}$ $p < 0.1$, * $p < 0.05$, ** $p < 0.01$, *** $p < 0.001$. Control variables include income per capita, population, occupancy rate, N.H. concentration, White and Black male population, political preference, and For-Profit facilities. Control group: Never treated. These results are obtained using Callaway and Sant'Anna (CS) estimation method. A simple aggregation scheme in column (1), calendar time (2), group time (3), and length of exposure (4). The results include year and county-fixed effects. Calendar time involves computing an average treatment effect for all individuals that are treated in period t and then averaging across all periods. Length of exposure to treatment is used to test whether there are dynamic treatment effects, similar to the event study. Group time combines with the group average treatment effect by the size of each group. In this setup "group" is defined by the period when units are first treated. The first scheme presented here is a simple overall aggregation of participating in the treatment.

Table A6: Medicaid Expansion on Nursing Home Residents Composition (2006-219)

	TWFE (1)	CS (2)
(A) Aggregate Nursing Home Residents: Black		
Medicaid Expansion	-0.34**	-0.13*
	(0.11)	(0.07)
Adjusted R^2	0.97	
Observations	25277	
(B) Aggregate Nursing Home Residents: Hispanic		
Medicaid Expansion	0.09	0.42^{+}
	(0.07)	(0.23)
Adjusted R^2	0.96	
Observations	23732	
(C) Aggregate Nursing Home Residents: White		
Medicaid Expansion	0.73***	0.65*
	(0.20)	(0.31)
Adjusted R^2	0.91	
Observations	29739	

Note: Standard errors are in parentheses. Significance is denoted as follows: $^{+}$ $p < 0.1$, * $p < 0.05$, ** $p < 0.01$, *** $p < 0.001$. Two-way Fixed Effects (TWFE) and the Callaway and Sant'Anna (CS) results are included. Control variables include income per capita, population, occupancy rate, N.H. concentration, White and Black male population, political preference, and For-Profit facilities. Control group: Never treated. Period: from 2006 to 2019.

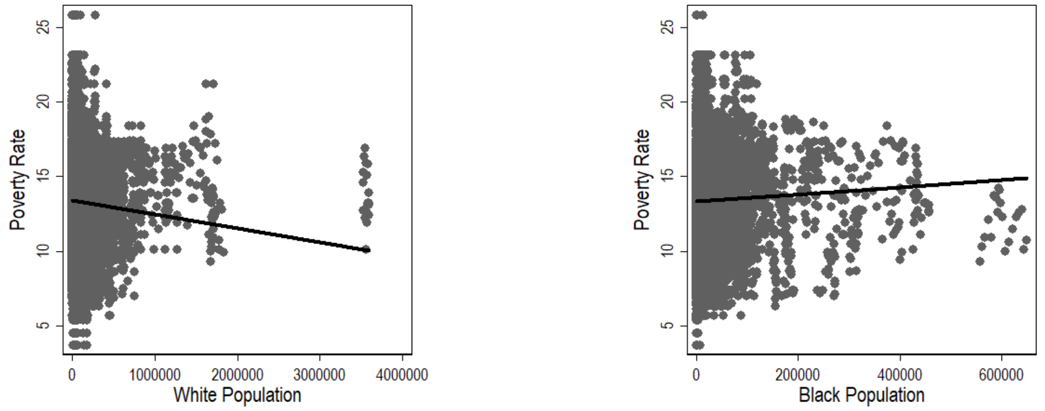

(a) White Population (b) Black Population

Figure A6: Correlation between Poverty Rate and Race

Note: This graph shows the correlation between poverty rates and population. It plots in panel (a) White and (b) Black populations. Data sources are detailed in section 2.

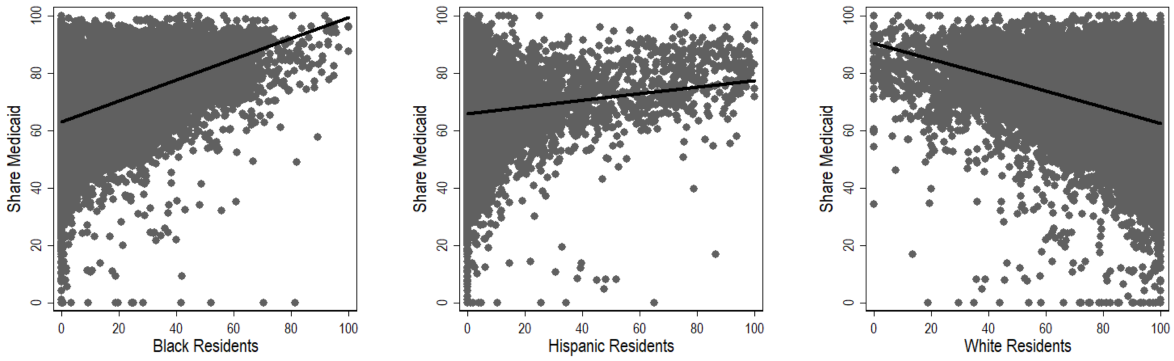

(a) Black Residents (b) Hispanic Residents (c) White Residents

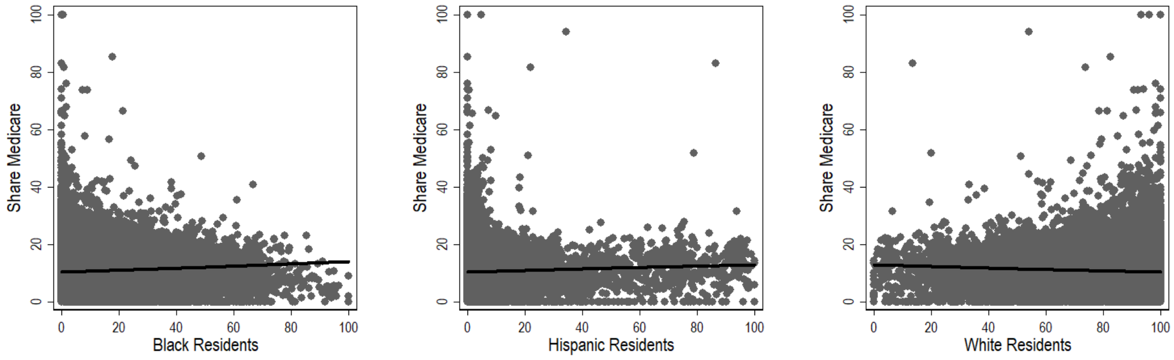

(d) Black Residents (e) Hispanic Residents (f) White Residents

Figure A7: Correlation between Medicaid/Medicare and Nursing Home Residents

Note: This graph shows the correlation between the share of residents whose primary support is Medicaid/Medicare and the aggregate number of residents in a nursing home by race/ethnicity.

Table A7: Classification of States by Poverty Rate and Income Inequality

**Poverty Rate**			**Income Inequality**		
High	Low		High	Low	
Alabama	Colorado	North Dakota	Arkansas	Alabama	Pennsylvania
Arizona	Connecticut	Ohio	California	Arizona	Rhode Island
Arkansas	Delaware	Oregon	Connecticut	Colorado	South Carolina
California	Hawaii	Pennsylvania	Florida	Delaware	Utah
Florida	Idaho	Rhode Island	Georgia	Hawaii	Vermont
Georgia	Illinois	South Dakota	Idaho	Indiana	Virginia
Kentucky	Indiana	Utah	Illinois	Iowa	Washington
Louisiana	Iowa	Vermont	Louisiana	Kansas	West Virginia
Mississippi	Kansas	Virginia	Massachusetts	Kentucky	Wisconsin
New Mexico	Maine	Washington	Mississippi	Maine	
New York	Maryland	Wisconsin	Montana	Maryland	
North Carolina	Massachusetts	Wyoming	Nevada	Michigan	
Oklahoma	Michigan		New Jersey	Minnesota	
South Carolina	Minnesota		New Mexico	Missouri	
Tennessee	Missouri		New York	Nebraska	
Texas	Montana		Oklahoma	New Hampshire	
West Virginia	Nebraska		South Dakota	North Carolina	
	Nevada		Tennessee	North Dakota	
	New Hampshire		Texas	Ohio	
	New Jersey		Wyoming	Oregon	

Note: The table classifies the U.S. states by high or low-income inequality. It is based on the author's calculations. The explanation of the classification is presented in section 3.

Table A8: Medicaid Expansion on Mechanisms: Classification by Poverty Rate

	TWFE		CS		TWFE		CS	
	Low	High	Low	High	Low	High	Low	High
	(A) P. Medicaid				(B) P. Medicare			
Medicaid Expansion	-0.67*	-0.33	-0.75*	-0.26	0.12	-0.02	-0.04	-0.04
	(0.34)	(0.45)	(0.34)	(0.44)	(0.20)	(0.23)	(0.19)	(0.24)
Adjusted R^2	0.69	0.62			0.65	0.57		
Observations	23758	21723			23758	21723		
	(C) P. Private				(D) T.Beds			
Medicaid Expansion	0.55^{+}	0.36	0.79*	0.31	3.86	5.49	-7.50	-11.41^{+}
	(0.31)	(0.39)	(0.33)	(0.39)	(3.90)	(5.64)	(7.19)	(6.76)
Adjusted R^2	0.78	0.61			1.00	1.00		
Observations	23758	21723			23758	21723		

Note: Standard errors are in parentheses. Significance is denoted: $^{+}$ $p < 0.1$, * $p < 0.05$, ** $p < 0.01$, *** $p < 0.001$. Two-way Fixed Effects (TWFE) and the Callaway and Sant'Anna (CS) results are included. The variables P.Medicaid in panel (1), P.Medicare in (2), P.Private in (3), and T. Beds in (4) were examined as dependent variables. Control variables include income per capita, population, occupancy rate, N.H. concentration, White and Black male population, political preference, and For-Profit facilities. The results include year and county-fixed effects. The dependent variables are defined in section 3.1.

Table A9: Effects on Nursing Home Race/Ethnic Composition with Mechanisms by Poverty Classification

	(1)				(2)				(3)			
	TWFE		CS		TWFE		CS		TWFE		CS	
	Low	High	Low	High	Low	High	Low	High	Low	High	Low	High
(A) Aggregate Nursing Home Residents: Black												
Medicaid Expansion	0.10	-0.70***	0.00	-0.38*	0.10	-0.71***	0.00	-0.47**	0.09	-0.72***	-0.02	-0.49**
	(0.08)	(0.20)	(0.08)	(0.19)	(0.08)	(0.20)	(0.08)	(0.18)	(0.08)	(0.20)	(0.09)	(0.16)
P. Medicaid	0.01**	0.02***										
	(0.00)	(0.01)										
P. Private					-0.01**	-0.01+						
					(0.00)	(0.01)						
T. Beds									-0.00**	0.00		
									(0.00)	(0.00)		
Adjusted R^2	0.96	0.95			0.96	0.95			0.96	0.95		
Observations	20941	19639			20941	19639			20941	19639		
(B) Aggregate Nursing Home Residents: White												
Medicaid Expansion	0.40	0.99**	0.62*	0.24	0.41	0.99**	0.62*	0.37	0.41+	1.00**	0.67*	0.52
	(0.25)	(0.33)	(0.29)	(0.35)	(0.25)	(0.33)	(0.31)	(0.34)	(0.25)	(0.33)	(0.29)	(0.34)
P. Medicaid	-0.03**	-0.02**										
	(0.01)	(0.01)										
P. Private					0.03**	0.03**						
					(0.01)	(0.01)						
T. Beds									0.00***	-0.00		
									(0.00)	(0.00)		
Adjusted R^2	0.84	0.92			0.84	0.92			0.84	0.92		
Observations	23500	21542			23500	21542			23500	21542		

Note: Standard errors are in parentheses. Significance is denoted: + $p < 0.1$, * $p < 0.05$, ** $p < 0.01$, *** $p < 0.001$. Two-way Fixed Effects (TWFE) and the Callaway and Sant'Anna (CS) results are included. Control variables include income per capita, population, occupancy rate, N.H. concentration, White and Black male population, political preference, and For-Profit facilities. Additional variables are included: in columns (1) P.Medicaid, (2) P.Private, and (3) T.Beds. These variables are defined in section 3.1. The results include year and county-fixed effects.

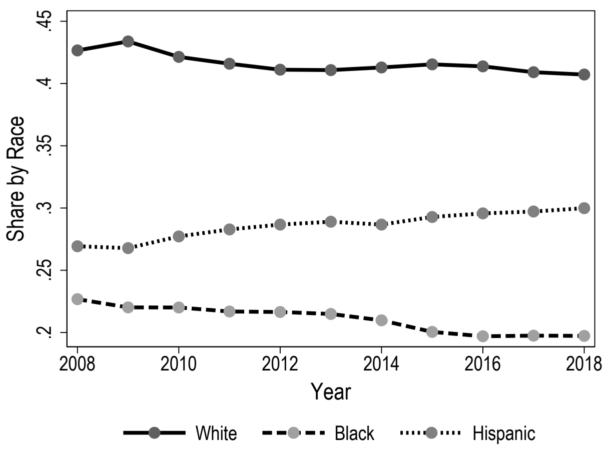

Figure A8: Medicaid Coverage Rates for Nonelderly by Race/Ethnicity

Note: This figure divides the Medicaid coverage rates by race/ethnicity from 2008 to 2018 for the U.S. average. The data used is obtained from KFF estimates based on the 2008-2019 American Community Survey.

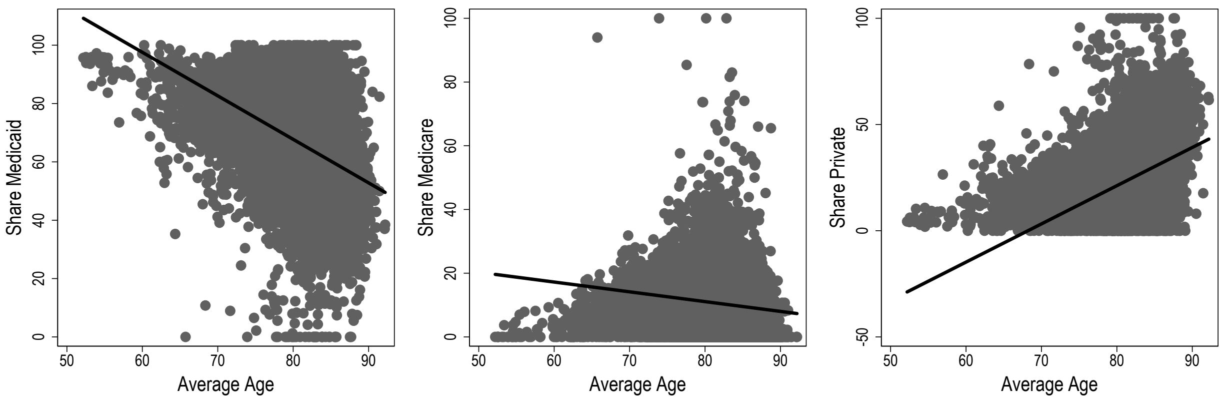

(a) Medicaid

(b) Medicare

(c) Private

Figure A9: Correlation between Form of Payment and Average Age

Note: This graph shows the correlation of average age with the share of Medicaid (a), Medicare (b), and Private (c) forms of payment in the United States.
